# A Novel Detector Based on Convolution Neural Networks for Multiscale SAR Ship Detection in Complex Background

**DOI:** 10.3390/s20092547

**Published:** 2020-04-30

**Authors:** Wenxin Dai, Yuqing Mao, Rongao Yuan, Yijing Liu, Xuemei Pu, Chuan Li

**Affiliations:** 1 College of Computer Science, Sichuan University, Chengdu 610065, China; 2017223045183@stu.scu.edu.cn (W.D.); rgyuan@stu.scu.edu.cn (R.Y.); liuyijing@scu.edu.cn (Y.L.); 2 College of Cybersecurity, Sichuan University, Chengdu 610065, China; maoyuqing@stu.scu.edu.cn; 3 College of Chemistry, Sichuan University, Chengdu 610065, China

**Keywords:** convolutional neural network (CNN), ship detection, synthetic aperture radar (SAR), multiscale and small ship detection, complex background

## Abstract

Convolution neural network (CNN)-based detectors have shown great performance on ship detections of synthetic aperture radar (SAR) images. However, the performance of current models has not been satisfactory enough for detecting multiscale ships and small-size ones in front of complex backgrounds. To address the problem, we propose a novel SAR ship detector based on CNN, which consist of three subnetworks: the Fusion Feature Extractor Network (FFEN), Region Proposal Network (RPN), and Refine Detection Network (RDN). Instead of using a single feature map, we fuse feature maps in bottom–up and top–down ways and generate proposals from each fused feature map in FFEN. Furthermore, we further merge features generated by the region-of-interest (RoI) pooling layer in RDN. Based on the feature representation strategy, the CNN framework constructed can significantly enhance the location and semantics information for the multiscale ships, in particular for the small ships. On the other hand, the residual block is introduced to increase the network depth, through which the detection precision could be further improved. The public SAR ship dataset (SSDD) and China Gaofen-3 satellite SAR image are used to validate the proposed method. Our method shows excellent performance for detecting the multiscale and small-size ships with respect to some competitive models and exhibits high potential in practical application.

## 1. Introduction

Synthetic aperture radar (SAR) can provide high-resolution images under all-weather and all-day conditions [1,2,3,4], thus playing an important role in marine monitoring and maritime traffic supervision [5,6,7,8]. Ship detections of the SAR images have attracted considerable interests [9,10,11,12,13], which usually consist of four steps: land masking [14], preprocessing, prescreening, and discrimination [15]. The purpose of the land masking is to eliminate adverse effects of the lands, while the preprocessing aims at improving the detection precision in subsequent stages. The prescreening step is used to locate candidate areas as ship region proposals. Among the prescreening methods, constant false alarm rate (CFAR) prescreening is the most widely used [10,16,17,18]. The discrimination is designed to eliminate false alarms and obtain real targets [19,20,21]. Traditional methods rely on hand-crafted features. Consequently, they are not promising for ship discrimination in front of complex backgrounds, which generally contain inshore or offshore locations (ship-like interferences, such as roofs, container piles, and so on), or distractions caused by sea clutter [14,15]. Therefore, it is urgent to develop new detection methods to improve the detection performance for the SAR ships. 

Convolution neural network can learn deep features from the data itself [22]. Its feature extraction performs much better than the hand-crafted one for target detections [23,24,25,26]. Thus, convolution neural network (CNN)-based detectors have been applied to detect ships in the SAR images. Among the CNN methods, Faster RCNN (F-RCNN) [27] based on the region proposal is a typical detection algorithm. F-RCNN consists of the shared convolution network used for extracting features, the region proposal network (RPN) for predicting candidate regions, and the detection network for classifying ship proposals and refining their spatial locations. In F-RCNN, RPN uses an anchor mechanism to generate the region proposal directly from the topmost feature map. However, the detection performance of the F-RCNN algorithms has not been satisfactory for the small-size ships with pixels less than 30 px [28]. Thus, Li et al. [29] proposed several strategies such as transfer learning and hard negative mining to improve the standard F-RCNN algorithm. They used CNN with five layers to detect the public SAR ship dataset (SSDD), which contains different-size ships covering offshore and inshore areas. Experimental results showed that the average precision of the improved F-RCNN is 78.8%, which is 8.7% higher than that of the previous one. Although the precision of the ship detection was improved to some extent, it is still not satisfactory. This may be attributed to the small number of CNN layers, the complex background of the SAR images, and the variable sizes of ships.

As known, the feature map from each layer has differences in semantic distinction and spatial resolution for CNN. Thus, CNN has a tradeoff between them [11]. In general, shallow layers of CNN have higher spatial resolutions than the other layers. Feature maps of intermediate layers are complementary with a passable resolution, while feature maps of high layers are abstract and semantic, which could distinguish target categories. Consequently, the shallow layers are more suitable for the location while the high layers are conducive to classification [30,31]. To deal with the detection of the variable-size ships, Zhao et al. [15] proposed a coupled CNN detector, which was based on an idea of fusion feature map from the Single Shot Detector (SSD) [32] algorithm. They used a VGG16 network with 16 convolution layers and merged the last three-layer feature maps to improve semantic information. Compared with the F-RCNN method, the average precision was improved from 71.3% to 79.5% for collected Gaofen-3 datasets, which contain many small and densely clustered ships. In addition, Ji et al. [8,11] proposed a multilayer fusion convolutional neural network for the SAR ship detection, in which three shallow layers were combined. Compared with the F-RCNN method, the detection precisions were improved from 73.9%/67.2% to 83.6%/87.3% on a collected Sentinal-1 dataset. Gui et al. [22] merged shallow layers and high layers (discarding intermediate layers) to detect multiscale objects, based on a light-head detector. They achieved 84.4% of detection precision for SSDD, which was 7.8% higher than that of F-RCNN under the same experimental configurations. These observations verify that the feature merging is beneficial for improving the detection performance of the multiscale ships. However, the detection results are not very satisfactory, which are desired to be further improved either for the feature fusion or for the model construction. 

Based on all the considerations above, we propose a novel SAR ship detection framework to identify the multiscale ships against complex backgrounds. In order to improve the detection performance of the multiscale ships, we also fuse feature maps in bottom–up and top–down forms, and we generate proposals from each fused feature map in order to make full use of the semantic information and the spatial one. Different from other related works, we change the convolution network to a residual learning framework [33] in order to further improve the detection performance and avoid overfitting of high network depth. In general, the RoI pooling layer extracts a fixed-length feature vector from the coarse region proposal generated by RPN in order to predict the target. As pointed out, the small size object lacks information for the location optimization and classification [11]. Thus, in order to improve their detection performance, we further merge each feature map generated by the RoI pooling layer to enhance the feature information, which is also different from the previous models with inclusion of the feature merging [8,11,22]. As expected, our experiments on the public SAR Ship Detection Dataset (SSDD) and the Chinese Gaofen-3 dataset show that the proposed framework could significantly improve the detection performance on the ship targets with different sizes in front of complex backgrounds.

The rest of this paper is organized as follows. Section 2 describes the framework of our method in detail. Section 3 introduces the datasets used in the work and the experimental results. The final section gives the conclusion.

## 2. Methodology

**Figure 1:**
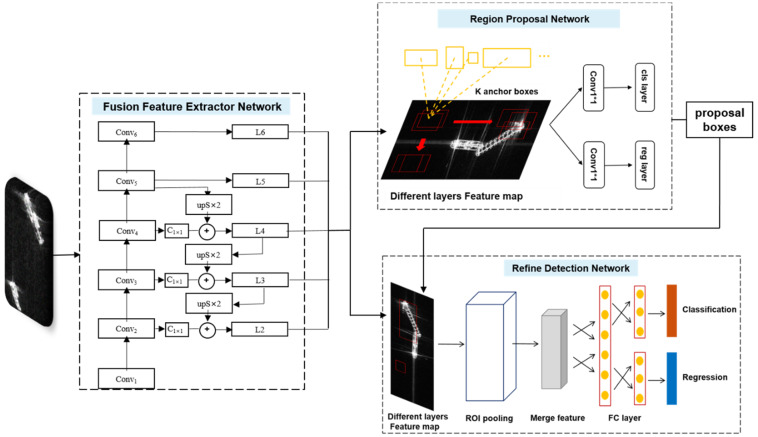
The architecture of our proposed method, which consists of the Fusion Feature Extractor Network (FFEN), Region Proposal Network (RPN), and Refine Detection Network (RDN).

Figure 1 illustrates the detailed architecture of our proposed method, including three subnetworks: Fusion Feature Extractor Network (FFEN), Region Proposal Network (RPN), and Refine Detection Network (RDN). Firstly, FFEN extracts features from the SAR images and fuses features through the bottom–up and top–down ways, which are shared by the following two subnetworks. Next, RPN is used to predict the region proposals at each feature fusion layer. Finally, RDN implements the target detection, based on the region proposals and the feature maps from FFEN. Detailed introductions for the three subnetworks are shown in the following sections. In addition, we also test the computational costs of the three subnetworks after the whole framework is constructed. 

### 2.1. Fusion Feature Extractor Network

**Figure 2:**
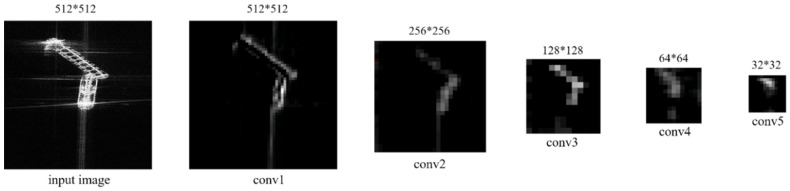
Visualization of feature maps from different convolution layers in VGG16, *convi* (*i* = 1, 2, 3, 4, 5) denotes different convolution layers from shallow to high.

As known, convolution neural networks are generally composed of multiple convolution layers and pooling layers, through which CNN can extract features from the input image. In order to reduce the number of parameters in the neural network, CNN always shrinks its feature maps after the convolutions by means of the max pooling operation. Herein, we take VGG16 for example to visualize the feature maps of different convolution layers. In Figure 2, *convi* (*i* = 1, 2, 3, 4, 5) denotes different convolution layers from shallow to high in VGG16. It can be seen that the shallow layers (*conv1* and *conv2*) present higher spatial resolutions but are scarce in the semantic information. One pixel on *conv1* almost corresponds to one pixel in the input image; thus, it is similar in size to the input image. After the pooling layer reduces the number of the training parameters and the dimension of the feature vectors from the convolution layers, the feature map will become small, thus showing lower resolution. As depicted by Figure 2, the feature semantic information of higher layers such as *conv4* and *conv5* is rich but abstract, in which one pixel corresponds to several pixels of the input image. Thus, the object location in the high layers is rough. Overall, the shallow layers can achieve more accurate location, and the high layers are conducive to classify in a wide range. Thus, we construct FFEN by fusing feature information of all the convolution layers in order to make full use of the semantic and spatial information.

Herein, we use the idea of Feature Pyramid Networks (FPN). Specifically, the structure includes bottom–up and top–down processes, as shown in the left side of Figure 1. In the bottom–up feedforward network, there are often many layers producing output maps with the same sizes, which are taken as one feature mapping layer. In total, we select such five feature mapping layers *Conv_i_* (*i* = 2, 3, 4, 5, 6), and *Conv_6_* is a stride two max-pooling of *Conv_5_*. The feature extracted from each feature mapping layer is the output of its last layer with strong semantic information. A top–down approach is adopted, which first undergoes a 1 × 1 convolutional layer (vide *C_1×1_* in Figure 1) to reduce the dimension of corresponding *Conv_i_* (*i* = 2, 3, 4), and uses the nearest neighbor up-sampling to up-sample the fused feature maps higher than it to its size. Then, the up-sampled map is merged with the corresponding bottom–up one, as shown in Figure 1. For example, the up-sampled map *Conv_5_* is merged with *Conv_4_*, which generates *L_4_*. Then, the up-sampled map *L_3_* is merged with *Conv_3_*, outputting *L_3_*. Finally, the fusion of the up-sampled map *L_3_* and *Conv_2_* generates *L_2_*. This process is iterated until the finest resolution map is obtained. In addition, a 3 × 3 convolution filter is appended to each fused feature map to generate the fusion feature mapping layer *L_i_* (*i* = 2, 3, 4, 5) so that the aliasing effect of the upper sampling could be reduced. Consequently, the merged feature mapping layer could enhance integrity of the location and semantics information, which is beneficial for the multiscale ship detection.

**Figure 3:**
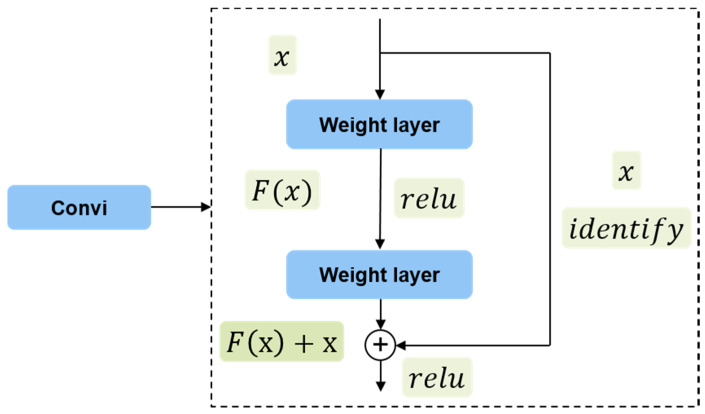
The shortcut connection of ResNet.

Ren et al. [34] pointed out that the CNN depth is very important to improve the performance of the feature representation. However, as the depth increases, the training of the network becomes difficult due to an explosion of parameters and disappearance of gradients, which leads to a drop in the precision of the network. To solve the problem, a residual learning depth network based on ResNet was proposed to ease the training process and improve the detection accuracy [34]. Instead of stacking convolution layers directly, ResNet connects these layers to fit a residual mapping. Formally, *x* denotes the input SAR image, and *H(x)* represents the underlying output mapping. We let the stacked nonlinear layers fit another mapping of *F(x)*:= *H(x) − x*. Then, the original mapping is recast into *F(x)* + *x.* The process could be realized by feedforward networks with shortcut connections, as shown in Figure 3. The shortcut connections do not add additional parameters and computational complexity. Based on the strategy, the entire network could propagate signals with more layers. Herein, ResNet50 is used as the residual network [34].

### 2.2. Region Proposal Network

**Figure 4:**
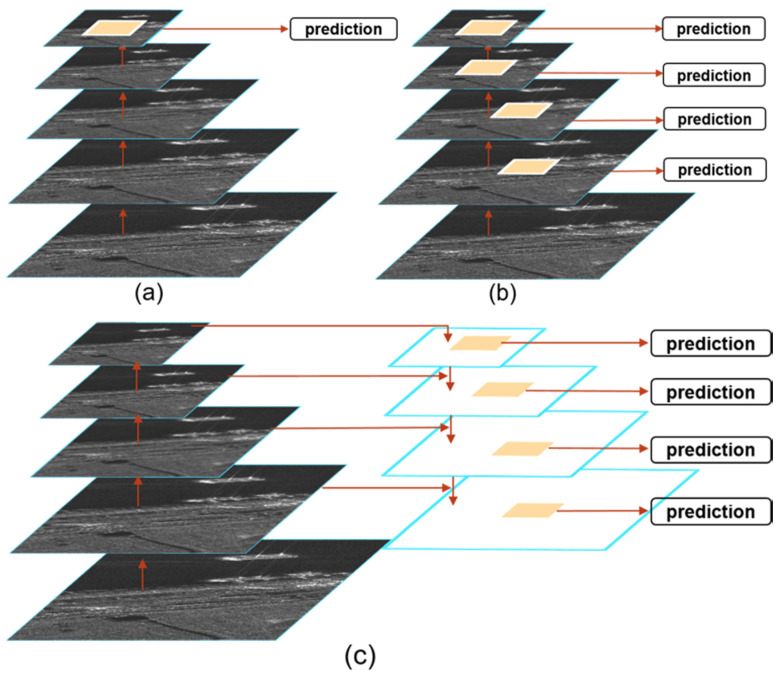
Different strategies for the multiscale detection. (**a**) Prediction from the top feature map such as Faster RCNN (F-RCNN); (**b**) Prediction from multiple feature maps such as Single Shot Detector (SSD); (**c**) RPN of our framework is a hierarchical prediction structure of feature fusion.

The region proposal network is utilized to classify the ships and the background in the SAR images and to generate coarse region proposals through using the fusion feature mapping layer *Li* (*i* = 2, 3, 4, 5, 6) provided by FFEN as inputs. The feature maps of different layers represent different feature semantic information and spatial resolutions. For the F-RCNN detector, only the top-level features of the network are used for prediction (see Figure 4a). This may be attributed to the fact that it cannot detect the multiscale ships well. Single Shot Detector (SSD) uses multiscale feature fusion to extract features from the middle and top layers for prediction, as shown in Figure 4b. Although these methods utilized the feature fusion, they ignored the low-level feature information, which is useful for the accurate location. Thus, in order to make full use of the feature semantic information, we design a hierarchical prediction structure of feature fusion, in which RPN is attached to each fusion feature map *Li* so that it could achieve high performance for the detection of the multiscale ships in the complex background, as shown in Figure 4c.

For RPN, we use anchors (a set of reference boxes, also called as region proposals) to measure the ship position and predict whether it is a ship target. The anchors are involved in multiple predefined scales and aspect ratios in order to cover ship targets of different scales. All the anchors have the same center points. We assign five different-scale (*Scale_i_* (*i* = 2, 3, 4, 5, 6) = {32 × 32, 64 × 64, 128 × 128, 256 × 256, 512 × 512}) anchors to each fusion feature mapping layer *L_i_* (*i* = 2, 3, 4, 5, 6). The aspect ratios of the anchors of the fusion feature mapping layers *L_i_* (*i* = 2, 3, 4, 5, 6) are {1:1, 1:2, 2:1}. Consequently, 15 (5 scales and 3 aspect ratios) anchors are generated for each *Li* (*i* = 2, 3, 4, 5, 6). As shown in Figure 1, these anchors are transmitted to the cls_layer and reg_layer in RPN (cls_layer for the ship target classification and reg_layer for the anchor regression). The cls_layer outputs 2K (K = 15) scores, which are used to estimate probability of the object for each proposal. The reg_layer has 4K outputs encoding coordinates of boxes. Since this stage produces a large number of coarse anchors and many of them overlap each other, we use non-maximum suppression (NMS) [35] to reduce the number of coarse anchors. The retention of the anchors is measured by Intersection-Over-Union (IoU) between each anchor and the corresponding ground-truth. IoU is generally defined as:

(1) $$\mathrm{IoU}=\;({Area}_{bbox}\cap {Area}_{gt})\;/\;({Area}_{bbox}\cup {Area}_{gt}\;)$$

where *Area_bbox_* and *Area_gt_* represent the prediction box and the ground-truth box, respectively. If the IoU of an anchor is higher than 0.7, it is considered as a positive anchor. An anchor with IoU less than 0.3 is taken as a negative anchor. The anchors with IoU in the range of 0.3–0.7 are ignored and do not participate in the training. For each image, we sample 512 anchors to train, in which a ratio of 1:1 is used for the sampled positive and negative anchors.

### 2.3. Refine Detection Network

As reflected by Figure 1, the Refine Detection Network (RDN) is the third stage of our algorithm framework, which uses the characteristics provided by FFEN and the coarse anchors of RPN as inputs. Its main function is to refine the coarse anchors and get the final prediction result. In RDN, the RoI pooling layer extracts a fixed-length feature vector with a 7 × 7 × 512 size from the coarse region proposal generated by RPN. In order to enhance the semantic information about the small-size objects, we further merge the features generated by the RoI pooling layer. Then, the merged features are fed back to the fully connected layers to obtain the final detection result, as shown by Figure 1. The impact of the feature merging in RDN will be evaluated in the following experiment section.

### 2.4. Computational Costs

Herein, we test the computational cost of the whole network. Table 1 shows the structure of ResNet-50, the number of parameters, and the multiply–add computational cost (MAC), which was derived from the 224 × 224 size of the input image block. The parameters and MAC of each layer are computed in terms of the configuration of each layer. Table 2 summarizes the MAC and the number of parameters for the three subnetworks (FFEN, RPN, and RDN). As shown in Table 2, our method requires 53 billion MAC and 260 million parameters for an iteration. Judged from MAC, the FFEN part is the least in the computing cost. The required times for training and testing mainly depend on the RPN and RDN parts. The result also indicates that the computing cost of FFEN with inclusion of ResNet-50 is not increased despite increasing the number of convolution layers.

## 3. Experiments and Results

In this section, experiments are carried out to evaluate the performance of the proposed method. First, we briefly describe the datasets used and experimental settings. Then, we used a standard dataset (the public synthetic aperture radar (SAR) ship detection dataset, SSDD) to evaluate the performance of the proposed framework. Finally, our model is further applied to the Gaofen-3 dataset (the first high-resolution civil SAR satellite in China) in order to test its robustness in practice. For the two types of datasets, our model is compared with some competitive methods reported and exhibits better performances.

### 3.1. Experimental Datasets and Settings

#### 3.1.1. Dataset Descriptions

**Figure 5:**
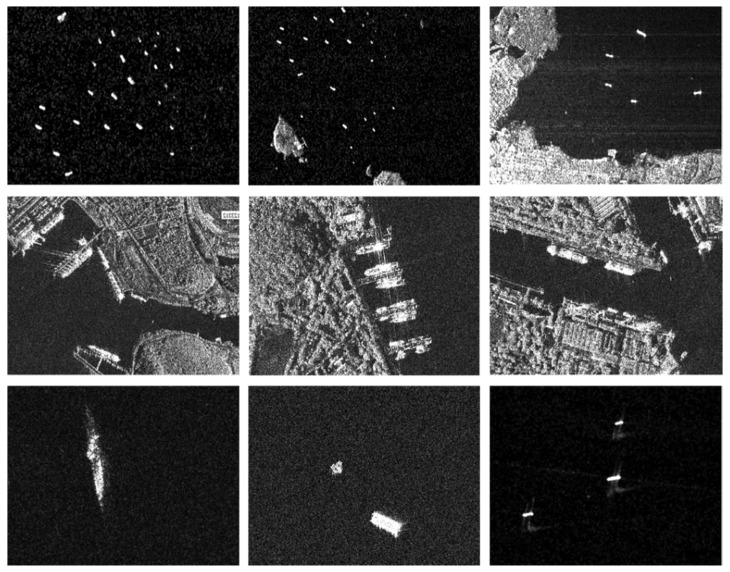
Some representative images of the SAR ship detection dataset (SSDD) involved in multiscale, near-shore, and small ships.

The public SAR Ship Detection Dataset (SSDD) [29] is used in the work, which follows a similar format to Pascal VOC [36]. SSDD includes SAR images collected from Radarsat-2, Terrasar-x, and Sentinel-1 [37] with resolutions ranging from 1 to 15 m and polarimetric modes of HH, HV, VV, and VH. Table 3 lists specific information of the ships in SSDD. In SSDD, there are a total of 1160 images and 2456 ships, and the average number of ships per image is 2.12. Statistics for the number of the ships and the images are shown in Table 4. We divide the dataset into three parts (training set, test set, and validation set) with the ratio of 7:2:1. Figure 5 representatively shows some images of SSDD. In addition, in order to further verify the robustness of our model in practice, we also use the SAR image taken from Ganfen-3 as one independent test set, which contains 102 ships with different sizes in a complex environment. Gaofen-3 is the first C-band multi-polarization SAR satellite developed by China, and its resolution could reach 1 m. The specific information of the Gaofen-3 dataset is listed in Table 5.

#### 3.1.2. Experimental Settings

All experiments are implemented using the deep learning framework Caffe [38] and executed on a PC with an Intel(R) Xeon(R) CPU E3-1230 v5 @ 3.40GHz, NVIDIA GTX-1080T GPU (12 GB memory), and the PC operating system is Ubuntu 16.04. We firstly use the pretraining model ResNet-50 to initialize our network. Then, we utilize the end-to-end training strategy to train our model, in which the gradient descent algorithm is used to update the network weight. A total of 40 k iterations are performed. The learning rate of the first 20,000 iterations is 0.001, and the learning rate of the last 20,000 iterations is 0.0001. The weight decay and momentum are set to be 0.0001 and 0.9, respectively. 

#### 3.1.3. Evaluation Metrics

In this work, we utilize three criteria widely used to quantitatively evaluate the detection performance. They are precision, recall, and F1-score. The precision measures the detection fraction of true positive samples in terms of Equation (2).

(2) $$\mathrm{precision}\;=\frac{TP}{TP+FP}$$

The recall measures fraction of positives over the number of ground-truths, which is defined by Equation (3)

(3) $$\mathrm{recall}\;=\frac{TP}{TP+FN}$$

Herein, TP, FN, and FP denote true positive, false negative, and false positive, respectively. In general, a detection result is considered to be a true positive if the overlap ratio of the IoU between a detected bounding box and a ground truth bounding box is greater than 0.5. Otherwise, the detection is considered as a false positive. IoU is generally defined by Equation (1) above.

As shown in Equation (4), the F1-score combines the precision and recall metrics as a single measure; thus, it could comprehensively evaluate the quality of the ship detection model:

(4) $$F1=2\times \frac{precision\times recall}{precision+recall}$$

### 3.2. Experiments on SSDD

#### 3.2.1. The Effect of the Number of Network Layers

As known, the depth of the convolution layers is associated with the detection precision. In order to observe the effect of the depth of the convolution layers, we test and compare three network depths (layer-5 (ZF [39]), layer-16 (VGG16 [40]), and layer-50 (ResNet-50 [34]). To eliminate the influence of other factors, we only change the network depth, not considering the other operations such as the feature fusion. Table 6 lists the detection precision, recall, and F1-score for the three types of network depths. It can be seen that the 50-layer (ResNet-50) model exhibits the best performance for recall, precision, and F1-score, indicating that the precision of SAR ship detection could be improved by increasing the depth of the network within the framework of the residual block. Thus, the 50-layer network is adopted in the subsequent experiments.

#### 3.2.2. The Effect of Feature Merging in RDN 

**Figure 6:**
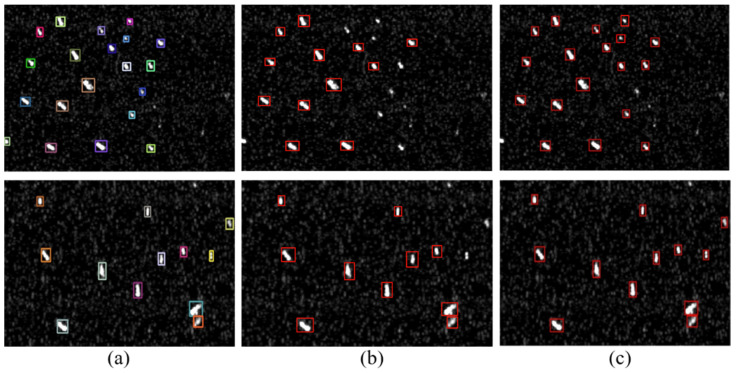
The detection results of the small size ships. (**a**) is the ground truth, (**b**) is the detection result of the model without merging features generated by the region-of-interest (RoI) pooling layer (called as no-merge model), (**c**) is the detection result of the model with the feature merging (called as merge model).

As mentioned above, the small-size object lacks information regarding the location optimization and the classification. Thus, in order to improve their detection performances, we fully merge the features generated by the RoI pooling layer and compare the results between the model with inclusion of the feature merging (labeled as the merge model) and one without the feature merging (labeled as the no-merge model). Table 7 lists their detection precisions, detection recalls, and F1-scores. It can be seen that the recall values of the two models are similar, but the precision and the F1-score of the merge model are higher than those of the no-merge model. Therefore, the feature merging in RDN could further improve the detection performance. In order to observe the impact of the feature merging on the detection performance of the small-size ships, we further check the number of small ships detected by the two models. There are 269 small ships in total for the test set. Herein, the target with less than 30 px is considered as the small-size ship [28]. The model without the feature merging could correctly identify 242 ships, while it is increased to 256 after merging the features. Figure 6 representatively displays the detection results of the two models. It is also observed that the merge model could identify more small-size SAR ships than the no-merge one. These observations confirm the efficacy of our fusing strategy in improving the detection of the small-size ships.

#### 3.2.3. Comparisons with Other Methods

To further evaluate the detection performance of our model, some competitive methods applied to SSDD are compared, including traditional a CFAR detector [41], Faster RCNN (F-RCNN) [27], Coupled-CNN_E_A [15], SSD [32], and a multilayer fusion light-head detector (MFLHD) [22]. These comparison results are shown in Table 8. Herein, we construct an improved CFAR based on the traditional two-parameter CFAR detector through combining a morphological filter and a density filter. The Faster RCNN method was reported to be a particularly influential detector, in which 16 convolution layers (VGG16) were used. Coupled-CNN_E_A and MFLHD are detectors specially designed to detect the multiscale ships in the SAR images, which exhibited good performances for the ship detection in the complex environment. SSD is a single-stage detector and it is faster than F-RCNN, which used anchor boxes to predict bounding boxes from multiple feature maps with different resolutions. In comparison, we used the choices laid out in the original papers as soon as possible.

It can be seen from Table 8 that the traditional CFAR exhibits the poorest performance for the multiscale ship detection in the complex environment, while our method significantly improves the detection performance compared with the other methods for the SSDD dataset, as evidenced by the precision, recall, and F1-score. In addition, Li et al. [29] used an improved F-RCNN to perform the ship detection for the SSDD dataset. In the work, they utilized AP to evaluate the detection performance, rather than the three evaluation metrics used in the work. In order to compare, we also calculate the AP value (89.4%), which is significantly higher than 78.8% reported by the work [29]. These comparisons above further confirm that our proposed method has excellent performance in the ship detection. 

**Figure 7:**
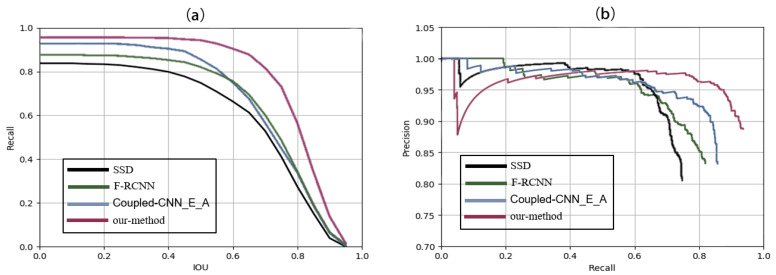
Performance curves for the four methods. (**a**) Recall vs. Intersection-Over-Union (IoU) curve, (**b**) Precision vs. recall curve.

On the other hand, we also compute recall and precision at different IoU ratios with the ground truth boxes for the four representative methods (SSD, F-RCNN, Coupled-CNN_E_A, and our method) in order to diagnose models, as shown in Figure 7. It can be seen from Figure 7a that the recall rate of each method decreases with increasing IoU. The recall rate of SSD detector is the lowest, and our method is superior to the other methods for recall-IoU. As reflected by Figure 7a, the recall values begin to drop when the IoU is higher than 0.5. Thus, it should be reasonable to set IoU to be 0.5 for calculating prediction results. In addition, Figure 7b further displays the precision-recall curve. A good model should possess high precision and high recall. However, the precision rate would present a drop when the recall rate is increased up to a point. As reflected by Figure 7b, the other three methods present sudden precision drops when the recall rate gets higher than 0.6, while our method begins to decrease when the recall rate is greater than 0.8. These observations further show the superiority of our model over the other three methods.

### 3.3. Robustness Testing on the GF-3 Dataset

#### 3.3.1. Detection Results and Comparisons

**Figure 8:**
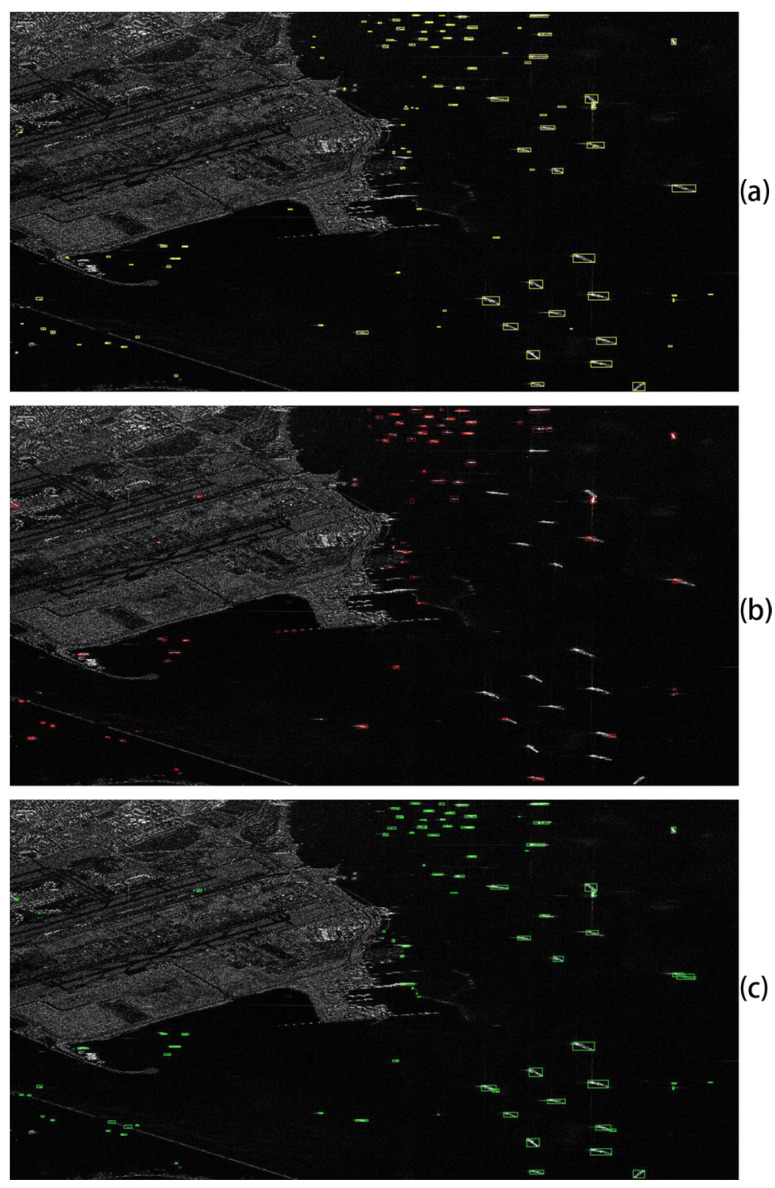
Detection results on the large GF3 SAR ship imagery (**a**) the ground truth; (**b**) detection results of F-RCNN; (**c**) detection results of our method. Yellow, red, and green rectangles represent the ground truth, the detection result of F-RCNN, and the detection result of our method, respectively.

As mentioned above, our method exhibits better performance on the SSDD dataset containing multiscale SAR ships. In order to further evaluate the application of our model in practice, it is applied to detect a large Ganfen-3 SAR image, which includes 102 ships with different sizes in the complicated environment (see Figure 8). Due to the large size of the whole Ganfen-3 SAR image, a 512 × 512 pixel sliding window is used without any overlapping. Similarly, the performance of our model is compared with the four representative detectors (CFAR, F-RCNN, Coupled-CNN_E_A, and SSD), as shown in Table 9. It can be seen that our method still exhibits better performance than the other methods for the independent GF-3 dataset. Figure 8 representatively shows the detection results from our method and F-RCNN, since F-RCNN has been recognized as a very influential detector. It is clear that our method almost detects all the ships on the ocean, including ships in offshore or inshore areas, while the F-RCNN method misses many ships. The result confirms that our method is effective for detecting the multiscale ships in practice.

#### 3.3.2. Analysis on Missing Ships and False Alarms

**Figure 9:**
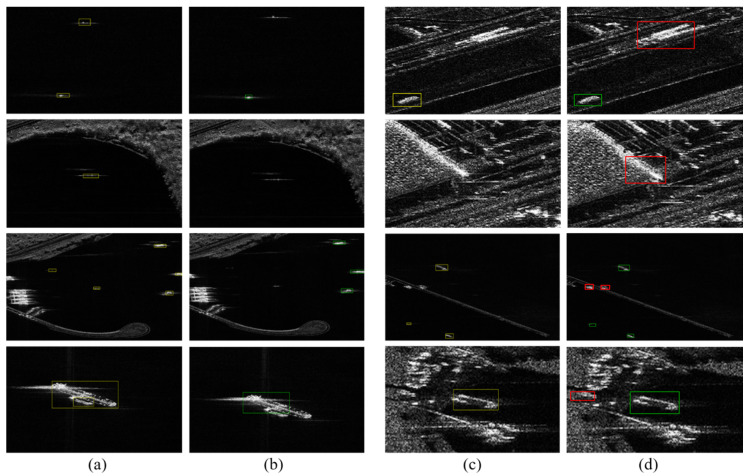
Some missing ships and false alarms for the detection result of the GF-3 synthetic aperture radar (SAR) image with our proposed method. (**a**) Ground truth; (**b**) detection result from our method with respect to (**a**); (**c**) ground truth; (**d**) detection result from our method with respect to (**c**). Yellow, green, and red rectangles denote the ground truth, the detection result of our method, the false alarm, respectively.

Although our method achieves excellent performance for the SSDD dataset and the GF-3 image, a few missing ships and false alarms still exist. For the GF-3 image with 102 ship targets, there are seven missing ships and nine false alarms. As can be seen from Figure 9a,b, some missing ships present very weak or low intensity, so that they would induce few responses on the shallow layers, in turn leading to them being missed. Recently, a new Perceptual Generative Adversarial Net-work (Perceptual GAN) model was proposed to improve the detection of small objects through narrowing the representation differences of the small objects from the large ones, rather than learning representations of all the objects at multiple scales [42]. The introduction of the perceptual GAN should be beneficial for detecting small size ships in the future. In addition, some ships side by side are detected to be one ship due to their close distances. It may be improved by modifying the method of non-maximum suppression (NMS) such as soft-NMS [43]. The method decays the detection scores of all other objects as a continuous function of their overlaps with the detection box so that no object is eliminated in the process. Besides these missing targets, some false alarms are also observed in our prediction results. They mainly come from some building facilities on land, some harbor facilities in the open ocean area, or near the coast, which are similar to ships in shape and intensity, as reflected by Figure 9c,d. For these false alarms, they may be ruled out with sea–land segmentation in image preprocessing or the addition of environmental information into the network.

## 4. Conclusions

In order to improve the detection performance for the multiscale ships and small-size ones in complex environments, we construct a novel CNN-based detector composed of a Fusion Feature Extractor Network (FFEN), Region Proposal Network (RPN), and Refine Detection Network (RDN). Instead of using a single feature map, we fuse feature maps in bottom–up and top–down ways and generate proposals from each fused feature map in FFEN. In addition, we further merge features generated by the region-of-interest (RoI) pooling layer in RDN. Based on the feature fusing strategy, rich location and semantics information could be obtained for the multiscale ships, in particular for the small-size ones. On the other hand, the residual block is introduced to FFEN in order to further improve the detection accuracy. Finally, the experimental results on the public SAR ship dataset (SSDD) and the Gaofen-3 satellite SAR image verify that our method could improve the detection performance of the multiscale and small-size ships in front of complex backgrounds. Compared to some competitive methods reported, our model exhibits better performance and high potential for practical applications.

## Figures and Tables

**Table 1:** The detailed structure, the number of parameters, and the multiply–add computational cost (MAC) for the FFEN network.

Name	Type	Stride	Output	Params	MAC
*Conv_1_*	[7×7,64] × 1	2	112 × 112 × 64	9.47 K	118.01 M
*Conv_2_*	$\left[\begin{array}{l} 1\times 1,64\\ 3\times 3,64\\ 1\times 1,256 \end{array}\right]$ × 3	2	112 × 112 × 64	9.47 K	118.01 M
*Conv* *_3_*	$\left[\begin{array}{l} 1\times 1,128\\ 3\times 3,128\\ 1\times 1,512 \end{array}\right]$ × 4	2	56 × 56 × 256	262.19 K	877.88 M
*Conv_4_*	$\left[\begin{array}{l} 1\times 1,256\\ 3\times 3,256\\ 1\times 1,1024 \end{array}\right]$ × 6	2	28 × 28 × 512	1154.1 K	1056.11 M
*Conv_5_*	$\left[\begin{array}{l} 1\times 1,512\\ 3\times 3,512\\ 1\times 1,2048 \end{array}\right]$ × 3	2	14 × 14 × 1024	7360.7 K	1389.24 M

**Table 2:** The number of parameters and MAC for each part of our network with the image size of 224 × 224 as the input.

	Params	MAC
FFEN	24.286 M	4201.04 M
RPN	7.499 M	6144.39 M
RDN	228.47 M	42736.8 M
Total	260.3 M	53 B

**Table 3:** The detailed information of the synthetic aperture radar ship dataset (SSDD) dataset.

Sensors	Resolution	Polarization	Ship	Position
Sentinel-1 RadarSat-2 TerraSAR-X	1–15 m	HH,VV VH,HV	Different size and material	In the sea and offshore

**Table 4:** Statistics for the number of ships and images.

NoS ^1^	1	2	3	4	5	6	7	8	9	10	11	12	13	14
NoI ^2^	725	183	89	47	45	16	15	8	4	11	5	3	3	0

^1^ NoS denotes the number of ships. ^2^ NoI denotes the number of images.

**Table 5:** Detailed information of the GF3 image.

Sensors	Resolution	Polarization	Ship	Position	Pixel	Imaging Time
GF-3	3M	HH	Different Size	Sea and offshore	Width: 29,986 Height: 15,648	17 July 2018

**Table 6:** Detection performances for three network depths, in which any feature merging is not considered.

Depth	Precision	Recall	F1-Score
5-layer	73%	84.4%	78%
16-layer	80.5%	86.4%	83.4%
50-layer	82.6%	87.1%	84.8%

**Table 7:** The effect of feature merging of RDN on detection performances, which include the feature merging of FFEN.

Methods	Precision	Recall	F1-Score
no-merge	86.9%	93.8%	90.2%
merge	89.9%	93.2%	91.5%

**Table 8:** Performance comparisons of several methods with our method for the SSDD dataset. The bold numbers denote the optimal values in each column. CFAR: constant false alarm rate.

Methods	Precision	Recall	F1-Score
CFAR	52.7%	64.2%	57.9%
F-RCNN	83.2%	81.9%	82.5%
Coupled-CNN_E_A	83.1%	85.7%	84.4%
SSD	80.4%	74.8%	77.5%
MFLHD [22] ^1^	87.5%	81.6%	84.4%
Our method	**89.9%**	**93.2%**	**91.5%**

^1^ The results come from Ref. [22].

**Table 9:** Performance comparisons of several methods with our method for the GF-3 dataset. The bold numbers denote the optimal values in each column.

Methods	Precision	Recall	F1-Score
CFAR	50.4%	62.9%	56.0%
F-RCNN	78.2%	83.5%	80.8%
Coupled-CNN_E_A	84.7%	82.2%	83.5%
SSD	79.2%	71.3%	75.1%
Our Method	**91.3%**	**93.1%**	**92.1%**
